# Virome Profiling, New Virus Identification and the Prevalence and Distribution of Viruses Infecting Chieh-Qua (*Benincasa hispida* Cogn. var. *chieh-qua* How) in China

**DOI:** 10.3390/v15061396

**Published:** 2023-06-19

**Authors:** Haiyan Che, Yuxin Ma, Yating Lin, Tuizi Feng, Daquan Luo, Haibo Long

**Affiliations:** 1 Environment and Plant Protection Institute, Chinese Academy of Tropical Agricultural Sciences, Haikou 571101, China; chehaiyan2012@126.com (H.C.); yuxin.ma@catas.cn (Y.M.); lytyazai@126.com (Y.L.); fengtuizi@163.com (T.F.); luodaquan@163.com (D.L.); 2 Key Laboratory of Pests Comprehensive Governance for Tropical Crops, Ministry of Agriculture and Rural Affairs, Haikou 571101, China; 3 Hainan Key Laboratory for Monitoring and Control of Tropical Agricultural Pests, Haikou 571101, China

**Keywords:** chieh-qua, high-throughput sequencing, *Orthotospovirus*, *Crinivirus*, *Potyvirus*, *Cucumovirus*, *Alphaendornavirus*, novel viruses, viral disease

## Abstract

The cucurbit vegetable chieh-qua (*Benincasa hispida* var. *chieh-qua* How) is an important crop in South China and southeast Asian countries. Viral diseases cause substantial loss of chieh-qua yield. To identify the viruses that affect chieh-qua in China, ribosomal RNA-depleted total RNA sequencing was performed using chieh-qua leaf samples with typical viral symptoms. The virome of chieh-qua comprises four known viruses (melon yellow spot virus (MYSV), cucurbit chlorotic yellows virus (CCYV), papaya ringspot virus (PRSV) and watermelon silver mottle virus (WSMoV) and two novel viruses: cucurbit chlorotic virus (CuCV) in the genus *Crinivirus* and chieh-qua endornavirus (CqEV) in the genus *Alphaendornavirus*. The complete genomes of the two novel viruses in chieh-qua and three other isolates of CuCV in pumpkin, watermelon and cucumber were determined and the recombination signals of pumpkin and watermelon isolates of CuCV were detected. A reverse transcriptase PCR indicated that the dominant viruses of chieh-qua in Hainan are MYSV (66.67%) and CCYV (55.56%), followed by CuCV (27.41%), WSMoV (7.41%), cucumber mosaic virus (8.15%), zucchini yellow mosaic virus (6.67%), PRSV (6.67%) and CqEV (35.56%). Our findings support diagnostic and prevalence studies of viruses infecting chieh-qua in China, enabling sustainable control strategies for cucurbit viruses worldwide.

## 1. Introduction

Chieh-qua (*Benincasa hispida* Cogn. var. *chieh-qua* How), also known as fuzzy gourd, hairy melon or moa qua, is a variety of wax gourd (*Benincasa hispida* (Thunb.) Cogn.) in the *Cucurbitaceae* family. As an important vegetable crop, chieh-qua is widely cultivated throughout the world for its immature fruit, especially in South China and southeast Asian countries [1]. In China in 2021, the planting area of *Benincasa hispida* was about 6 million acres, with a yield of 58 million tons [2]. Chieh-qua is mainly planted in Guangdong, Guangxi and Hainan provinces in Southern China and the planting area has gradually spread from south to north China with increasing consumer demand [3,4,5].

However, with the expansion of cultivation, various diseases have arisen, especially viral diseases, which have been a major factor restricting the yield and quality of chieh-qua in China. Diverse virus-like symptoms, including mosaic, interveinal chlorosis, mottling and leaf deformation, are frequently observed in chieh-qua; however, there has been limited research on sporadic chieh-qua samples and only four viruses have been reported: zucchini tigre mosaic virus (ZTMV, genus *Potyvirus*), zucchini yellow mosaic virus (ZYMV, genus *Potyvirus*), papaya ringspot virus (PRSV, genus *Potyvirus*) and cucumber mosaic virus (CMV, genus *Cucumovirus*) [6,7,8]. ZYMV, PRSV and CMV have been frequently identified in cucurbit vegetables in China [9], whereas ZTMV has only been discovered in China in recent years, with a potential risk to infect more cucurbit crops in the field [9,10]. Recently, Nong [11] investigated both wax gourd and chieh-qua plants in Guangdong Province, China, and they discovered the above four viruses as well as six other viruses: cucurbit aphid-borne yellows virus (CABYV), melon aphid-borne yellows virus (MABYV), squash leaf curl China virus (SLCCNV), cucumber green mottle mosaic virus (CGMMV), watermelon silver mottle virus (WSMoV) and tobacco stripe virus (TSV). Among them, WSMoV was considered the dominant virus in the gourd population. Nevertheless, deep virome profiling and an extensive investigation of the virus diversity, occurrence and distribution on chieh-qua plants in China are still lacking.

The present study aimed to investigate in depth the viruses causing chieh-qua viral disease in China. For this, a ribosomal RNA (rRNA)-depleted total RNA method was used to construct the virome profiles of symptomatic chieh-qua samples. This approach identified four known viruses (MYSV, cucurbit chlorotic yellows virus (CCYV), PRSV and WSMoV) and two novel viruses in the genus *Crinivirus* and *Endornavirus*, whose complete genomes and natural hosts within gourd vegetables were also determined. Furthermore, an extensive virus screen was conducted on over two hundred samples collected from various plantations in Hainan province from 2021 to 2022. This study provided comprehensive information on the virus diversity of chieh-qua in China, the prevalence and geographical spread of viruses infecting chieh-qua and the dominant viruses in different plantations.

## 2. Materials and Methods

### 2.1. Plant Materials

**Figure 1:**
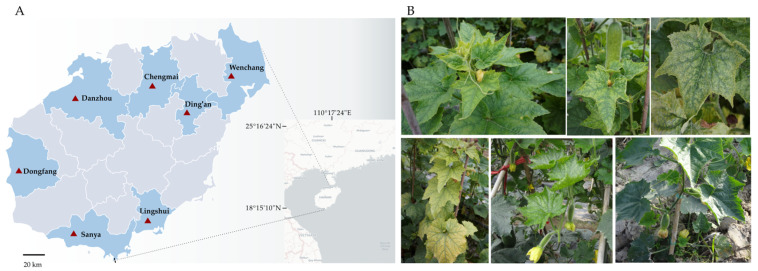
The cucurbit vegetable sampling sites in Hainan Province, China, and various virus-like symptoms in chieh-qua. (**A**) The cucurbit vegetable sampling sites in Hainan Province, China. (**B**) Various virus-like symptoms in chieh-qua. (Upper left) interveinal chlorosis and deformation. (Upper middle), interveinal chlorosis and mottling. (Upper right), mottling, mosaic and interveinal chlorosis. (Lower left) interveinal chlorosis and yellowing. (Lower middle), mosaic and interveinal chlorosis, and (lower right), healthy plant.

A total of 135 chieh-qua samples showing virus-like symptoms were collected for the prevalence survey of viruses infecting chieh-qua. In total, 98 samples from other cucurbit vegetables showing virus-like symptoms, including 23 pumpkin (*Cucurbita moschata*) samples, 26 watermelon (*Citrullus lanatus*) samples and 49 cucumber (*Cucumis sativus*) samples, were collected to determine whether the potential new viruses occur in other cucurbit vegetables. The above 223 symptomatic samples and 28 asymptomatic chieh-qua samples were collected from 7 main cucurbit vegetable cultivation regions (Chengmai, Wenchang, Ding’an, Lingshui, Sanya, Dongfang and Danzhou) in Hainan province from 2021 to 2022 (Figure 1A).

### 2.2. Nucleic Acid Preparation and HTS

Leaves from five individual chieh-qua plants (numbered CS-1, CS-15, CS-16, CS-37 and CS-78) representing the distinct symptoms (Figure 1B) were pooled together in equal amounts to form a composite sample (PL-1) for downstream total RNA extraction and HTS. Total RNAs were extracted using the TRIzol Reagent (Invitrogen, Carlsbad, CA, USA) following the supplier’s guidelines. A Nanodrop ND-1000 spectrophotometer (Thermo Fisher Scientific Inc., Waltham, MA, USA) and gel electrophoresis were used to monitor the quality and quantity of the RNA. Its concentration was determined using a Qubit^®^ 2.0 Fluorometer (Life Technologies, Carlsbad, CA, USA) and its integrity was evaluated using a 2100 Bioanalyzer (Agilent, Santa Clara, CA, USA). An Epicentre Ribo-Zero Gold Kit (Illumina, San Diego, CA, USA) was used to deplete ribosomal RNAs from the total RNA samples. An Illumina TruSeq^®^ RNA Sample Prep Kit (Illumina, San Diego, CA, USA) was then used to construct a cDNA library from the ribosomal RNA-depleted RNA. The cDNA library was then subjected to HTS using the PE150 sequencing platform on an Illumina NovaSeq 6000 sequencer (Illumina, San Diego, CA, USA), carried out by LC-Bio Technology Co., Ltd. (Hangzhou, China).

### 2.3. Data Processing and Virus Annotation

The raw reads from rRNA-depleted total RNA sequencing were trimmed to remove low-quality and adapter sequences using fastp [12]. The genome of *Benincasa hispida* Cogn. var. *chieh-qua* How has not been published; therefore, the genome sequence of Wax gourd (*Benincasa hispida*) (https://ftp.ncbi.nlm.nih.gov/genomes/all/GCF/009/727/055/GCF_009727055.1_ASM972705v1/, accessed on 13 January 2021) was used as the reference to eliminate host-derived sequences using HISAT v2.1.0 [13] for dataset size reduction. Trinity v2.13.3 [14] was used to de novo assemble the unmapped reads, which were then annotated according to BLASTn and BLASTx [15] searches against the nonredundant nucleotide (nt) and protein (nr) databases in GenBank [16], employing conservative cut-off e-values of 10^−4^ and 10^−6^, respectively.

### 2.4. RT-PCR Protocol

An RNAPrep Pure Plant Kit (TianGen, Beijing, China) was used to extract the total RNA from individual plants, which was reverse transcribed into cDNA using random hexamers and TransScript One-Step gDNA Removal and cDNA Synthesis SuperMix (TransGen, Beijing, China) following the supplier’s recommendations. The 2×Rapid Master Mix (Vazyme, Nanjing, China) was used to carry out subsequent PCR reactions using specific virus detection primers (Table S1). These primers were designed against the assembled contig sequences using Primer3 [17]. The reaction conditions for virus-specific PCR were: heating at 95 °C for 2 min; followed by 35 cycles of 95 °C for 45 s, 53 °C or 55 °C for 45 s and 72 °C for 45 s; followed by holding at 72 °C for 10 min and then holding at 4 °C until the analysis. A TIANgel Midi Purification kit (TianGen, Beijing, China) was used to purify the target amplicons, which were either sequenced directly or first ligated into vector pMD18-T (Takara, Kusatsu, Japan). The recombinant vectors were transformed into *Escherichia coli* DH5α competent cells (Takara, Kusatsu, Japan). We then selected three individual clones of each amplicon that had inserts of the expected size, which were sequenced in both directions using Sanger sequencing, carried out at BGI (Shenzhen, China).

### 2.5. Recovery of the Complete Genome and Characterization of the Newly Identified Viruses

Following confirmation of their virus status, the whole genomes of the newly discovered viruses were amplified using 2× Phanta Flash Master Mix (Vazyme, Nanjing, China) with various pairs of virus-specific primers (Table S2). The sequence of the novel crinivirus of *Closteroviridae* was obtained with complete 5′ and 3′ untranslated regions (UTRs). A SMARTer^®^ RACE 5′/3′ kit with SeqAmp DNA Polymerase (Takara, San Jose, CA, USA) was used to determine the 5′ UTR sequence of the novel crinivirus. Poly(A) Polymerase (Takara, Kusatsu, Japan) was then used to add additional ATPs to the 3′ terminus of the viral genome lacking a poly(A) tail following the supplier’s guidelines. Subsequently, the 3′ terminal sequence was completed using an anchored oligo (dT) primer (5′-TGTGTTGGGTGTGTTTGGTTTTTTTTTTTTTTT-3′) to synthesize the cDNA from the poly(A)-tailed RNA virus, followed by amplification using the primer anchored-dt-rev (5′-TGTGTTGGGTGTGTTTGG-3′) together with a gene-specific primer.

For genomic characterization, the web tool ORFfinder (https://www.ncbi.nlm.nih.gov/orffinder/, accessed on 2 December 2022) was used to predict the open reading frames (ORFs) and the Conserved Domain Database (CDD, https://www.ncbi.nlm.nih.gov/Structure/cdd/wrpsb.cgi, accessed on 2 December 2022) was used to analyze conserved motifs and domains. Transmembrane domains were predicted using DeepTMHMM [18]. Pairwise comparisons were calculated from CLUSTALW multiple alignments and MEGA 11 [19] was used to construct neighbor-joining (NJ) phylogenetic trees with 1000 bootstraps.

### 2.6. Recombination Analysis

We scanned the complete genome sequence alignment file for potential recombination events involving five cucurbit chlorotic virus (CuCV) isolates (cucumber-SY, pumpkin-DF, chieh-qua-CM, watermelon-LS and Thailand) and the CYSDV isolates available in the NCBI database using the recombination analysis software SimPlot version 3.5.1 [20] and the Recombination Detection Program v.4.43 (RDP4) [21]. Except for the selection of the linear genome option, each RDP4 analysis used the default settings. We considered only the events detected by a minimum of five of the seven detection methods (RDP, GENECONV, Bootscan, MaxChi, Chimaera, 3Seq and SiScan), which had at least three *p*-values < 10^−6^ and combination scores above 0.6. Events with a recombination score between 0.4 and 0.6 were considered fairly likely.

## 3. Results

### 3.1. HTS Output, Discovery of Viruses and HTS Validation

The rRNA-depleted total RNA sequencing of sample PL-1 produced 84,840,500 raw reads (81,484,200 clean reads), whose Q20 and Q30 values were >94%. A total of 77,465,294 (95.07%) of the reads mapped to the wax gourd reference genome. *De novo* assembly of the remaining 4,018,906 (4.93%) unmapped reads produced 23,111 contigs (lengths = 201–15,020 nt). BLASTn and BLASTx analyses identified 31 contigs corresponding to viruses of the *Closteroviridae* family (12 contigs, length ranging from 904 to 8886 nt), *Tospoviridae* (14 contigs, length ranging from 251 to 8902 nt), *Potyviridae* (3 contigs, length ranging from 270 to 10,320 nt) and *Endornaviridae* (2 contigs, with lengths of 14,990 and 15,020 nt) (Table 1). According to the generally low level of the amino acid sequence identity between the contigs and those of the known viruses in the NCBI database, we initially assumed that the chieh-qua composite sample (PL-1) comprised at least six viruses: MYSV, CCYV, WSMoV, PRSV, a novel crinivirus and a novel alphaendornavirus.

To confirm that the putative viruses were not artefacts of HTS sequencing, seven primer pairs (Table S1) were designed based on the assembled contigs for individual RT-PCR detection on all five plant samples used for HTS and the resultant amplicons were sequenced. The results confirmed that the five samples tested positive for one to six of the six viruses (Table S3) and sequences of these amplicons showed 99–100% identity with the sequences obtained by HTS. CuCV, MYSV and chieh-qua endornavirus (CqEV) were identified in chieh-qua for the first time.

### 3.2. Recovery of the Complete Genomes and Characterization of a Potential New Crinivirus in the Family Closteroviridae

**Figure 2:**
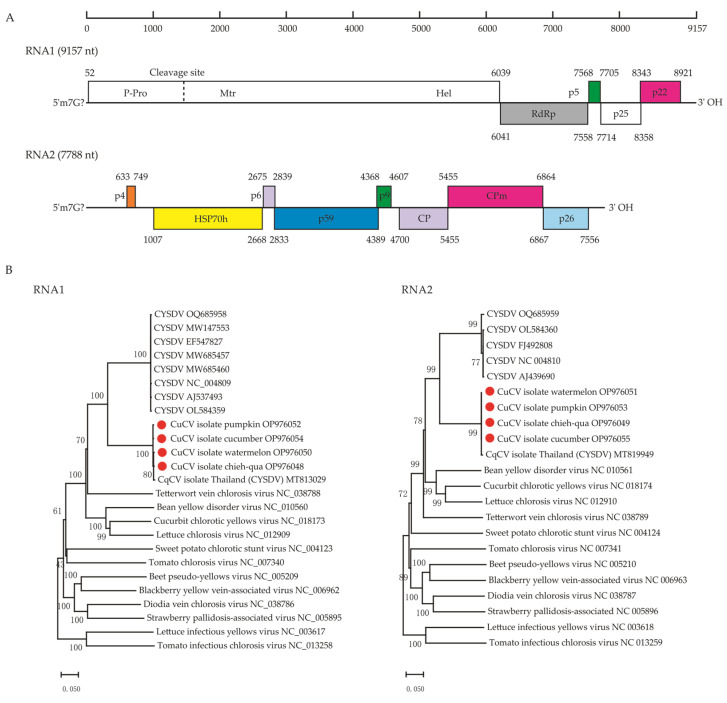
Cucurbit chlorotic virus (CuCV) genomic structure and phylogeny. (**A**) Schematic diagram of the CuCV genomic organization. The numbers below diagrams are the genomic nucleotide (nt) positions. Boxes represent the predicted open reading frames (ORFs), with indicated nt coordinates. Different colors represent the conserved motifs, domains and viral proteins within the ORFs: a viral methyltransferase domain (Mtr, pfam01660), viral helicase domain (Hel, pfam 01443), an RNA-dependent RNA polymerase (RdRp), heat-shock-protein-70-like protein (HSP70h), coat protein (CP), minor coat protein (CPm). Predicted proteins with unknown functions are also shown: a 5 kDa protein (p5), a 25 kDa protein (p25), a 22 kDa protein (p22) in CuCV RNA1 and a 4 kDa protein (p4), a 6 kDa protein (p6), a 59 kDa protein (p59), a 9 kDa protein (p9), a 26 kDa protein (p26) in CuCV RNA2. (**B**), Neighbor-joining (NJ) phylogenetic trees based on the complete nucleotide sequences of RNA1 (Left) and RNA2 (Right) segments of the representative members of the genus *Crinivirus*, family *Closteroviridae*. CYSDV, cucurbit yellow stunting disorder virus. The newly identified viruses in this study are indicated by red dots.

The entire genomes of the new crinivirus, CuCV, in chieh-qua were 9157 nt for RNA1 and 7788 nt for RNA2, whose poly(A) tails were of an undefined length (Figure 2A). To better understand the genomic sequence diversity of CuCV in different natural hosts, we also obtained the complete or near-complete genomes of CuCV from other cucurbit vegetables (pumpkin, watermelon and cucumber) (Table 2). The four CuCV isolates shared a high nt identity for the RNA1 (98.32–99.33%) and RNA2 (99.68–99.83%) genomic segments (Table 3).

BLASTn analyses revealed that CuCV and a so-called CYSDV Thailand isolate (MT813029 and MT819949) available in the GenBank database are molecularly identified as the same virus species. This conclusion is based on their complete genomes sharing more than 98% nucleotide identity (with 99% coverage). However, they exhibit significant divergence from other CYSDV isolates, with nucleotide identities ranging from 70.25% to 74.05% (coverage of 67% to 77%, e-value = 0.0) and a shared amino acid identity of 74.1% for the coat protein (Table S4). These findings challenge the existing nomenclature for the CYSDV Thailand isolate (MT813029 and MT819949), suggesting a misclassification. Through sequence comparison, phylogenetic analysis and literature research, we have determined that the CuCV identified in this study, along with the so-called CYSDV isolates from Thailand, potentially represent a distinct and divergent species separate from known CYSDV members (Figure 2B, Table S4).

The genomic organization of the characterized virus (Figure 2A) was very similar to those of members of the genus *Crinivirus* [22]. The RNA1 genomic segment contains five predicted ORFs, which encode a 1996 aa polyprotein (ORF1a, nt 52–6039, 229.83 kDa), RNA-dependent RNA polymerase (RdRp) (ORF1b, nt 6041–7558, 505 aa, 59.11 kDa; expressed from ORF1a via a +1 frame shift) and three proteins of unknown function (p5, p25 and p22). Based on similarities with other members of the genus *Crinivirus*, a putative P-Pro domain [23] (P-Pro, nt 52–1491) was predicted in the N-terminal of ORF1a, which has two conserved catalytic amino acids residues (Cys-412 and His-461), while the putative cleavage sites are between Gly-480 and Val-481. Viral methyltransferase (Mtr, nt 1705–2637, pfam 01660) domains, viral helicase (Hel, nt 5146–5937, pfam 01443) domains and two transmembrane domains between residues 1369 to 1379 and 1383 to 1394 were also predicted in ORF1a. The 5′- and 3′- UTRs of RNA1 are 51 and 237 nt, respectively.

RNA2 comprises eight predicted ORFs flanked by a 5′-UTR of 632 nt and a 3′-UTR of 231 nt, namely p4 (nt 633–749, 38aa, 4.5 kDa), a heat-shock-protein-70-like protein (HSP70h, nt 1007–2668, 553 aa, 62.4 kDa), p6 (nt 2675–2839, 54 aa, 6.62 kDa), p59 (nt 2833–4389, 518 aa, 59.67 kDa), p9 (nt 4368–4607, 79 aa, 9.44 kDa), coat protein (CP, nt 4700–5455, 251 aa, 28.80 kDa), minor CP (CPm, nt 5455–6864, 469 aa, 54.38 kDa) and p26 (nt 6867–7556, 229 aa, 26.89 kDa). Neither the p5 in RNA1 nor p4 in RNA2 have any homologs in the database. The seven nucleotides (ACATGGG) at the 5′-UTR of RNAs 1 and 2 are identical and although this feature is common in criniviruses [24], this sequence is otherwise unique.

### 3.3. The Genetic Variability and Recombination of CuCV Isolates from Cucurbit Vegetables

Phylogenetic relationships based on the RdRp and HSP70 protein sequences of representative members of *Crinivirus* in the *Closteroviridae* family revealed that four CuCV isolates (chieh-qua, cucumber, pumpkin and watermelon) in this study and the so-called CYSDV isolate from Thailand were consistently placed in a branch distinct from other CYSDV members (Figure S1). Although the RdRp and HSP70h proteins of CuCV shared the highest aa identities, of 90.10% and 83.91%, with those of CYSDV (Table S4), the CP shared only a 74.1% aa identity with CYSDV, which satisfies the accepted molecular criteria for species demarcation in the *Closteroviridae* family of <75% aa identity for the RdRp, HSP70h and CP genes [25].

**Figure 3:**
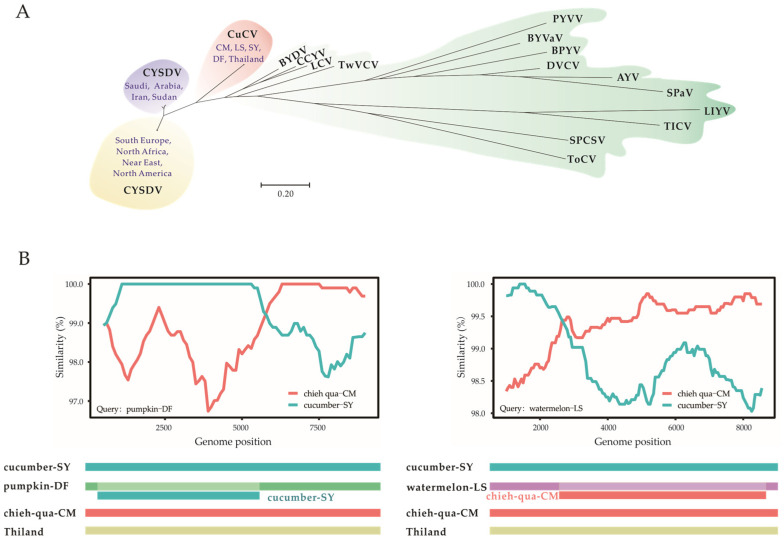
(**A**) The neighbor-joining (NJ) phylogenetic trees constructed based on the coat protein gene nucleotide sequences of cucurbit chlorotic virus (CuCV), cucurbit yellow stunting disorder virus (CYSDV) and representative members of the genus *Crinivirus*, family *Closteroviridae*. (**B**) Recombination analyses of five CuCV isolates using the recombination detection program Simplot and RDP4. Abbreviations and accession numbers for the viruses are AYV (abutilon yellows virus, AY422070), BPYV (beet pseudoyellows virus, NC_005210), BYDV (bean yellow disorder virus, NC_010561), BYVaV (blackberry yellow vein-associated virus, NC_006963), CCYV (cucurbit chlorotic yellows virus, NC_018174), CuCV (OP976049, OP976051, OP976053, OP976055, MT819949), CYSDV (AJ439690, AJ243000, AY730779, DQ903105-DQ903111, EF210558, EF210560, EF210561, EF210559, FJ492808, HG939523, KC677625, KC677626, KC469990-KC470000, KX768875, LT992903-LT992905, JF340435, JN083790, LT992890-LT992902, OL584360, OQ685959, NC_004810, MW685458, MW685459), DVCV (diodia vein chlorosis virus, NC_038787), LCV (lettuce chlorosis virus, NC_012910), LIYV (lettuce infectious yellows virus, NC_003618), PYVV (potato yellow vein virus, YP_054421), SPaV (strawberry pallidosis-associated virus, NC_005896), SPCSV (sweet potato chlorotic stunt virus, NC_004124), TICV (tomato infectious chlorosis virus, NC_013259), ToCV (tomato chlorosis virus, NC_007341), TwVCV (tetterwort vein chlorosis virus, NC_038789).

The phylogenetic reconstruction of the CP genes of 51 available CYSDV isolates, 5 CuCV isolates (cucumber-SY, Pumpkin-DF, watermelon-LS, chieh-qua-CM and Thailand) and the formally identified criniviruses showed that the CuCV isolates were located in a distinct cluster away from the CYSDV clusters, which, similar to the results of previous studies [26], was separated into two groups (Figure 3A). The two groups of CYSDV were located in (I) the Mediterranean Basin (South Europe, North Africa and the Near East) and North America and (II) the Middle East (Iran, Sudan and Arabia). No significant evidence of geographical association or host structure was observed in the population.

The Simplot and RDP software detected the two recombinants of CuCV isolate pumpkin-DF and watermelon-LS, the potential parents of which were CuCV isolate chieh-qua-CM and cucumber-SY (Figure 3B). The recombination break points in the CuCV isolate pumpkin-DF started at nt 370 and terminated at nt 5334, with the highest probability value of 10^−32^ according to the 3Seq method and a high recombination score of 0.815, which targeted the region encoding the replication-associated protein. The break points of the watermelon-LS isolate started at nt 2170 and terminated at nt 8686, with the highest probability value of 10^−12^ according to the SiScan method and a recombination score of 0.526 (Figure 3B). No significant recombination signals were detected between CuCV local isolates and the Thailand isolate or between any of the CuCV isolates and CYSDV isolates.

### 3.4. A New Alphaendornavirus in Family Endornaviridae

**Figure 4:**
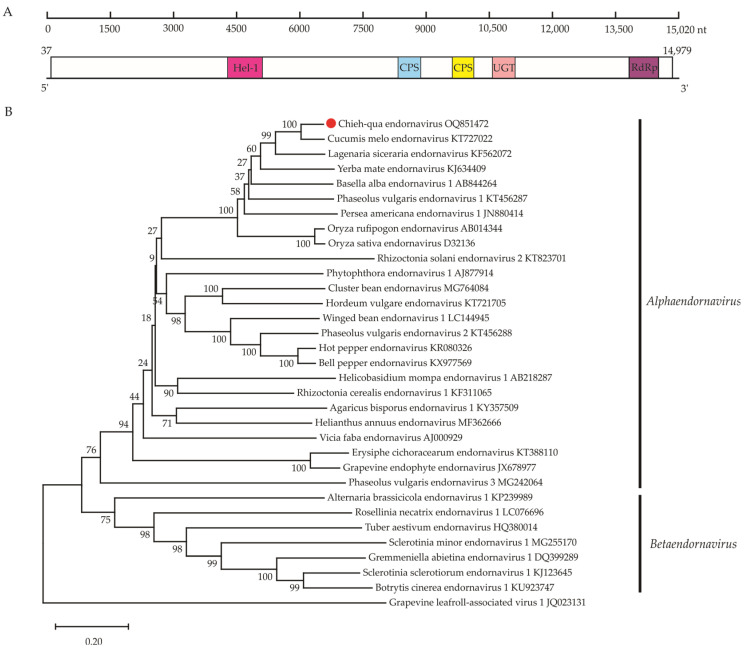
Genome organization and phylogeny of chieh-qua endornavirus (CqEV). (**A**) Schematic diagram showing the CqEV genomic organization: The numbers under the diagram are the nucleotide (nt) positions in the genome. Boxes indicate the predicted open reading frames (ORFs), with nt coordinates indicated below. Different colors indicate conserved domains: viral helicase 1 domain (Hel-1, pfam01443), two capsular polysaccharide synthesis protein domains (CPS, pfam05704), a UDP-glycosyltransferases domain (UGT, cd03784) and an RNA-dependent RNA polymerase domain (RdRp, cd23255). (**B**) The neighbor-joining (NJ) phylogenetic trees constructed based on the amino acid sequences of the RNA-dependent RNA polymerase domains of the representative *Endornaviridae* members with grapevine-leafroll-associated virus 1 as an outgroup member. The newly identified viruse in this study is indicated by red dots.

The novel alphaendornavirus, provisionally named chieh-qua endornavirus (CqEV) has a monopartite genome of ~15,020 nt based on the long scaffolds that were assembled from the rRNA-depleted total RNA sequencing, which lacks 5′- and 3′-terminal sequences (GenBank accession number OQ851472). Similar to other endornaviruses, the CqEV genome has only a single ORF encoding a 4981 aa polyprotein (estimated molecular mass = 573.13 kDa; encoded by nt 34–14,979) (Figure 4A). A conserved domain search detected a putative viral helicase 1 domain (Hel-1, aa 1438–1639, pfam01443), two capsular polysaccharide synthesis protein domains (CPS, aa 2772–2955, aa 3193–3321, pfam05704), a UDP-glycosyltransferases domain (UGT, aa 3709–3843, cd03784) and an RdRp domain (aa 4612–4848, cd23255). As expected, BlastN and BlastX analyses showed that cucumis melo endornavirus (CmEV) was most closely related to the endornaviral sequences.

To ascertain the relationship between CqEV and other members of the *Alphaendornavirus*, phylogenetic analysis of RdRP protein sequences from different endornaviruses (Figure 3B) revealed that CqEV consistently clustered with viruses in the genus *Alphaendornavirus* and formed a sister branch to CmEV (KT727022). Furthermore, the genome sequence of CqEV was compared with those of the 24 reported formal species in the genus *Alphaendornavirus*. The results indicated that CqEV is most closely related to CmEV (KT727022), sharing a 65.41% nt identity (Table S5). This fulfills the species demarcation criteria of overall nucleotide sequence identity below 75% [27]; therefore, the new virus belongs to a new species of the genus *Alphaendornavirus* in the family *Endornaviridae*.

### 3.5. The Prevalence of Viruses Infecting Chieh-Qua

**Figure 5:**
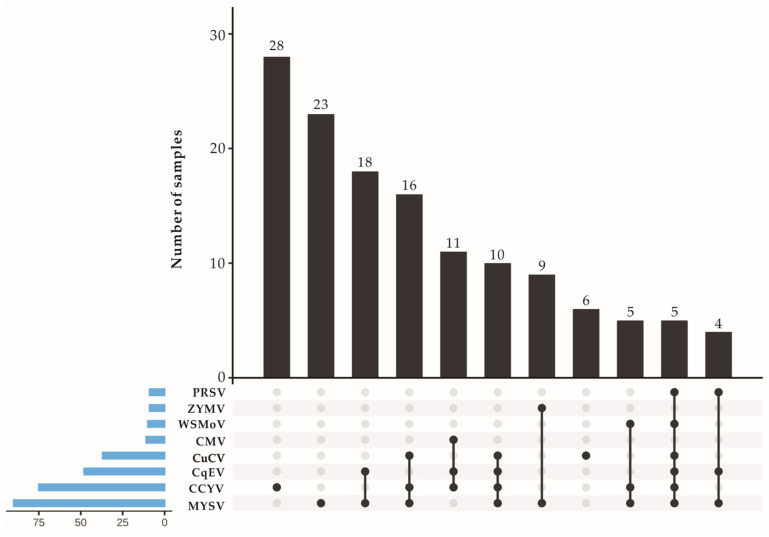
Virus infection types and the number of samples corresponding to each infection type in chieh-qua (this figure was created using EVenn [28]). Abbreviations for the viruses are PRSV (papaya ringspot virus), ZYMV (zucchini yellow mosaic virus), WSMoV (watermelon silver mottle virus), CMV (cucumber mosaic virus), CuCV (cucurbit chlorotic virus), CqEV (chieh-qua endornavirus), CCYV (cucurbit chlorotic yellows virus) and MYSV (melon yellow spot virus).

We examined 135 chieh-qua leaf samples from 7 counties (Table 3) to ascertain the distribution and incidence of the 6 viruses discovered by HTS and 3 viruses (i.e., CMV, ZYMV and ZTMV) reported for chieh-qua. RT-PCR and electrophoretic analyses indicated that MYSV, CCYV, CqEV and CuCV could be detected in all sampling sites. MYSV had the highest prevalence of 66.67% (90/135) and its incidence rate was 100% in samples from 4 sampled counties (Ding’an, Lingshui, Sanya, Dongfang). CCYV had a high prevalence of 55.56% (75/135). The new alphaendornavirus, CqEV, had a prevalence of 35.56% (48/135), with a high incidence of 92.86% and 100% in the Ding’an and Donfang regions, respectively. The new crinivirus, CuCV, had a prevalence of 27.41% (37/135) and was detected in samples from Chengmai, Wenchang, Ding’an, Lingshui and Sanya. The incidence of WSMoV, PRSV, ZYMV and CMV was generally low in most of the sites sampled (Table 3). MYSV and CCYV might be the main viruses infecting chieh-qua in Hainan province. Among the 135 samples, CqEV, WSMoV and PRSV were never detected to singly infect chieh-qua. A total of 78 (57.78%) samples were co-infected by multiple viruses with 8 types of co-infection, and 57 samples were detected as positive for 1 specific virus (Figure 5). Among the 28 asymptomatic chieh-qua samples collected from Chengmai in Hainan province in 2022, 9 were positive for CqEV according to RT-PCR, while all the samples were negative for CuCV.

To further explore the occurrence of the potential new crinivirus (CuCV) and the new endogenousvirus (CqEV) in different cucurbit vegetables, 98 symptomatic samples of 4 cucurbit vegetables were collected from 7 counties of Hainan. A total of 16 samples were CuCV positive by RT-PCR, including samples from pumpkin (4/23), watermelon (4/26) and cucumber (8/49) (Table S6). No CqEV was found in any of these 98 samples (Table S6), which indicated that CqEV is an endogenousvirus unique to chieh-qua.

## 4. Discussion

In this study, a putative novel crinivirus was identified and molecularly characterized. The closest relative of CuCV appears to be CYSDV, a *Bemisia-tabaci*-transmitted crinivirus causing extensive infections of cucurbit crops in many warm and temperate production areas [29,30,31,32,33]. The taxonomy of CuCV was first determined based on the low complete genome identity (65–69%) with CYSDV isolates compared to the 99% intraspecific similarity of CYSDV isolates (Tables S7 and S8), despite the extensive and discontinuous geographical distribution and different years of collection. However, CuCV and CYSDV share a high identity for the conserved RdRp and HSP70 proteins, which is frequently observed for other criniviruses, for example, amino acid similarities of 76.63–85.74% were discovered between diodia vein chlorosis virus (DVCV) and strawberry-pallidosis-associated virus (SPaV), bean yellow disorder virus (BYDV) and lettuce chlorosis virus (LCV) and tetterwort vein chlorosis virus (TwVCV) and BYDV (Table S9). In addition, the CP and all the other predicted proteins shared similarities of <75% at the AA level and at nt level with those of CYSDV (Table S10), which is far lower than the CYSDV intraspecific protein similarity. CuCV and CYSDV have a slightly different genomic arrangement. 5′- UTR of RNA1 and RNA2 of CuCV are approximately 1/2 of those of CYSDV in length and shared no significant nucleotide identities (Table S6). The 5′- end of CYSDV RNA 2 encodes a putative p4.9 protein that overlaps with HSP70h; however, the corresponding region of the CuCV genome encodes a unique p4 protein located distally from HSP70h. This evidence indicates the taxonomy of CuCV, a putative novel crinivirus in the family *Closteroviridae*.

Five CuCV isolates (four from this study and one from Thailand) from different natural hosts (chieh-qua, pumpkin, watermelon, cucumber and melon) showed generally limited spatial and temporal sequence variability, which is also found in some other viruses in the family *Closteroviridae* [33,34,35,36]. Although we identified significant recombination signals on the isolates from pumpkin and watermelon that appeared to be derived from chieh-qua and cucumber-hosted isolates, further validation is needed because the current biological data of the hosts and the geographical information cannot completely explain these recombination events.

In this study, a strong association between CuCV and specific symptoms was suggested because CuCV was found exclusively in symptomatic cucurbit samples but not in any of the 28 asymptomatic samples, which aligns with the results reported by Krishnan et al. [37] where CuCV (referred to as CYSDV in this article) was detected in all 16 symptomatic cucurbits samples from India but not in any of the asymptomatic samples. The statistical analysis results indicated that the specific symptoms caused by CuCV on cucurbit plants are potentially interveinal chlorosis, which is also in line with the study reported in India that CuCV causes interveinal chlorosis followed by bright yellowing in cucurbits (bitter gourd, cucumber and watermelon) [37].

CuCV is the fourth crinivirus reported to infect cucurbit plants in addition to CYSDV, CCYV and beet pseudoyellows virus (BPYV) [35,36]. So far, CuCV has been discovered in various cucurbits (melons, cucumbers, bitter melons, pumpkin, chieh-qua and watermelons) in Thailand, India and China, which indicates the potential broad host range of CuCV and the risk of a widespread outbreak of this virus. In addition, it has been suggested that CuCV is transmitted by whiteflies, like other criniviruses [38], thus further research is required to determine its biological, pathogenic and phytosanitary risks.

CqEV is the first endornavirus reported to infect chieh-qua. No CqEV was found as a single infection in symptomatic samples and 9 out of 28 asymptomatic samples were positive for CqEV, which indicated that CqEV is in a symbiotic relationship with chieh-qua causing no severe symptoms, similar to most plant endornaviruses, with the exception of *Vicia faba* endornavirus [39,40,41]. CqEV was only found in chieh-qua and not in cucumber, pumpkin and watermelon, which indicated that CqEV has a narrow host range. Except for cucumis melo endornavirus, which has the ability to infect three distinct genera in its family [42], the majority of endornaviruses have been identified in only one host species or, in some cases, in a limited number of closely related species within the same genus. Certain endornaviruses display an even narrower host range, only associating with specific genotypes or varieties of a particular host species, as is the case with phaseolus vulgaris endornavirus 1 (PvEV-1) and phaseolus vulgaris endornavirus 2 (PvEV-2) [43]. Although the percentage of plants containing endornavirus sequences is high (~8%) [44], the nature of their relationship with their host plants remains unclear.

In this study, chieh-qua is for the first time reported as the natural host of CuCV, MYSV and CqEV. Complex mix infection was commonly found in chieh-qua, though 57 samples were “single infected”. The virus-induced symptoms cannot differentiate between single or multiple infections, since the single-infected samples might have been infected with other unknown viruses besides the tested ones. Among the identified viruses, MYSV and CCYV were the most prevalent in this study and they were also reported to cause great losses in cucurbit vegetable production in many countries [45,46,47,48,49]. Further large-scale and in-depth investigations are needed concerning the virus diversity, epidemiology and evolution for the effective prevention and control of disease outbreaks in cucurbit and other plants.

## Figures and Tables

**Table 1:** Statistics for rRNA-depleted total RNA sequencing output of chieh-qua sample PL-1 by BLASTx analysis.

Family	Genus	Virus (Acc. No. of Protein)	No. of Contigs	Length Range (nt)	Identity Range (%)
*Closteroviridae*	*Crinivirus*	cucurbit yellow stunting disorder virus (NP_851566, NP_851572, NP_851576 and NP_851578)	5	1328–8886	66.1–83.9
*Closteroviridae*	*Crinivirus*	cucurbit chlorotic yellows virus (QFI57123, QFI57127, QFI57128, QFI57130 and YP_006522423)	7	904–8611	99.6–100
*Tospoviridae*	*Orthotospovirus*	watermelon silver mottle virus (QKX94930, NP_620752, NP_620767, NP_620770 and NP_620771)	6	465–7584	94.4–98.5
*Tospoviridae*	*Orthotospovirus*	melon yellow spot virus (YP_717933, YP_717935, QIG37604, QIG37605 and QIG37606)	8	251–8902	94.2–100
*Potyviridae*	*Potyvirus*	papaya ringspot virus (NP_056758 and BBK60930)	3	270–10,320	91.2–95.5
*Endornaviridae*	*Alphaendornavirus*	cucumis melo endornavirus (QIZ03212)	2	14,990–15,020	64–64.2

**Table 2:** The cucurbit chlorotic virus (CuCV) isolates used in this study.

Isolate	Host	Symptoms	Origin	Accession Numbers (RNA1, RNA2)
Chieh-qua-CM	*Benincasa hispida* Cogn. var. *chieh-qua* How	Chlorotic, mottling	Chengmai, Hainan	OP976048, OP976049
Watermelon-LS	*Cucurbita moschata*	Chlorotic, mosaic	Lingshui, Hainan	OP976050, OP976051
Pumpkin-DF	*Citrullus lanatus*	Chlorotic, mosaic	Dongfang, Hainan	OP976052, OP976053
Cucumber-SY	*Cucumis sativus*	Chlorotic, mosaic	Sanyan, Hainan	OP976054, OP976055

**Table 3:** Occurrence of viruses infecting chieh-qua in different plantations in Hainan Province, China.

Location	Percentage of Positive Samples (No. of Virus Infected Samples/Total No. of Plants Analyzed)							
	MYSV	CCYV	CqEV	CuCV	WSMoV	PRSV	CMV	ZYMV
Chengmai	36.11 (13/36)	33.33 (12/36)	22.22 (8/36)	33.33 (12/36)	5.56 (2/36)	5.56 (2/36)	0 (0/36)	2.78 (1/36)
Wenchang	30.43 (7/23)	69.57 (16/23)	21.74 (5/23)	13.04 (3/23)	0 (0/23)	0 (0/23)	8.7 (2/23)	0 (0/23)
Ding’an	100 (14/14)	57.14 (8/14)	92.86 (13/14)	28.57 (4/14)	0 (0/14)	7.14 (1/14)	28.57 (4/14)	0 (0/14)
Lingshui	100 (9/9)	61.54 (8/13)	33.33(3/9)	100 (9/9)	88.89 (8/9)	33.33 (3/9)	0 (0/14)	11.11 (1/9)
Sanya	100 (18/18)	66.67 (12/18)	16.67 (3/18)	50 (9/18)	0 (0/18)	11.11(2/18)	11.11(2/18)	11.11 (2/18)
Dongfang	100 (13/13)	23.53 (4/17)	100(13/13)	0 (0/13)	0 (0/13)	7.69 (1/13)	0 (0/13)	0 (0/13)
Danzhou	72.73 (16/22)	68.18 (15/22)	13.64 (3/22)	0 (0/22)	0 (0/22)	0 (0/22)	13.64 (3/22)	22.73 (5/22)
Total	66.67 (90/135)	55.56 (75/135)	35.56 (48/135)	27.41(37/135)	7.41 (10/135)	6.67 (9/135)	8.15(11/135)	6.67 (9/135)

Abbreviations for the viruses are MYSV (melon yellow spot virus), CCYV (cucurbit chlorotic yellows virus), CqEV (chieh-qua endornavirus), CuCV (cucurbit chlorotic virus), WSMoV (watermelon silver mottle virus), PRSV (papaya ringspot virus), CMV (cucumber mosaic virus) and ZYMV (zucchini yellow mosaic virus).

## Data Availability

The data presented in this study are available from the corresponding author upon reasonable request.

## Supplementary Materials

The following supporting information can be downloaded at: https://www.mdpi.com/article/10.3390/v15061396/s1, Figure S1: The neighbor-joining (NJ) phylogenetic trees constructed on the RNA-dependent RNA polymerase (RdRp) and heat shock protein 70 (HSP70) gene nucleotide sequences of cucurbit chlorotic virus (CuCV), cucurbit yellow stunting disorder virus (CYSDV) and the representative members of the genus Crinivirus, family Closteroviridae; Table S1: Specific primers used to confirm the presence of the virus in the original plants and to survey virus prevalence; Table S2: Primers used to complete the genomes of the new viruses; Table S3: RT-PCR confirmation of the presence of the virus in individual plants; Table S4: The percent amino acid identity RdRP, HSP70, and CP between the cucurbit chlorotic virus (CuCV) and other unclassified and formal members in the genus *Crinivirus* of the family *Closteroviridae*; Table S5: Overall nucleotide sequence identity (%) between chieh-qua endornavirus (CqEV) and members of different species in the genus *Alphaendornavirus* of the family *Endornaviridae*; Table S6: Occurrence of cucurbit chlorotic virus (CuCV) in different hosts; Table S7: The pairwise nucleotide identities (%) of genome RNA1 among cucurbit chlorotic virus (CuCV) virus and cucurbit yellow stunting disorder virus (CYSDV) isolates available in the NCBI database; Table S8: The pairwise nucleotide identities (%) of genome RNA2 among cucurbit chlorotic virus (CuCV) virus and cucurbit yellow stunting disorder virus (CYSDV) isolates available in the NCBI database; Table S9: The percent amino acid identity of RdRp (lower left) and HSP70h (upper right) among the formal species in the genus *Crinivirus* of the family *Closteroviridae*; Table S10. Percentages of the nucleotide (Nt) and deduced protein sequence (AA) identities between CuCV (RNA1: OP976048, OP976050, OP976052, OP976054, MT813029; RNA2: OP976049, OP976051, OP976053, OP976055 and MT819949) and CYSDV (RNA1: MW147553, EF547827, MW685457, MW685460, AY242077, AJ537493 and OL584359; RNA2: FJ492808, AY242078, MW685458, MW685459, OL584360 and AJ439690) available in the NCBI database.
