# Supplementary material for: Virome Profiling, New Virus Identification and the Prevalence and Distribution of Viruses Infecting Chieh-Qua (Benincasa hispida Cogn. var. chieh-qua How) in China
Source: Viruses. 2023 Jun 19;15(6):1396. doi: 10.3390/v15061396 (PMC10301065; doi:10.3390/v15061396)
Supplement: Supplementary file 1 [file viruses-15-01396-s001.zip › Figure S1.pdf]

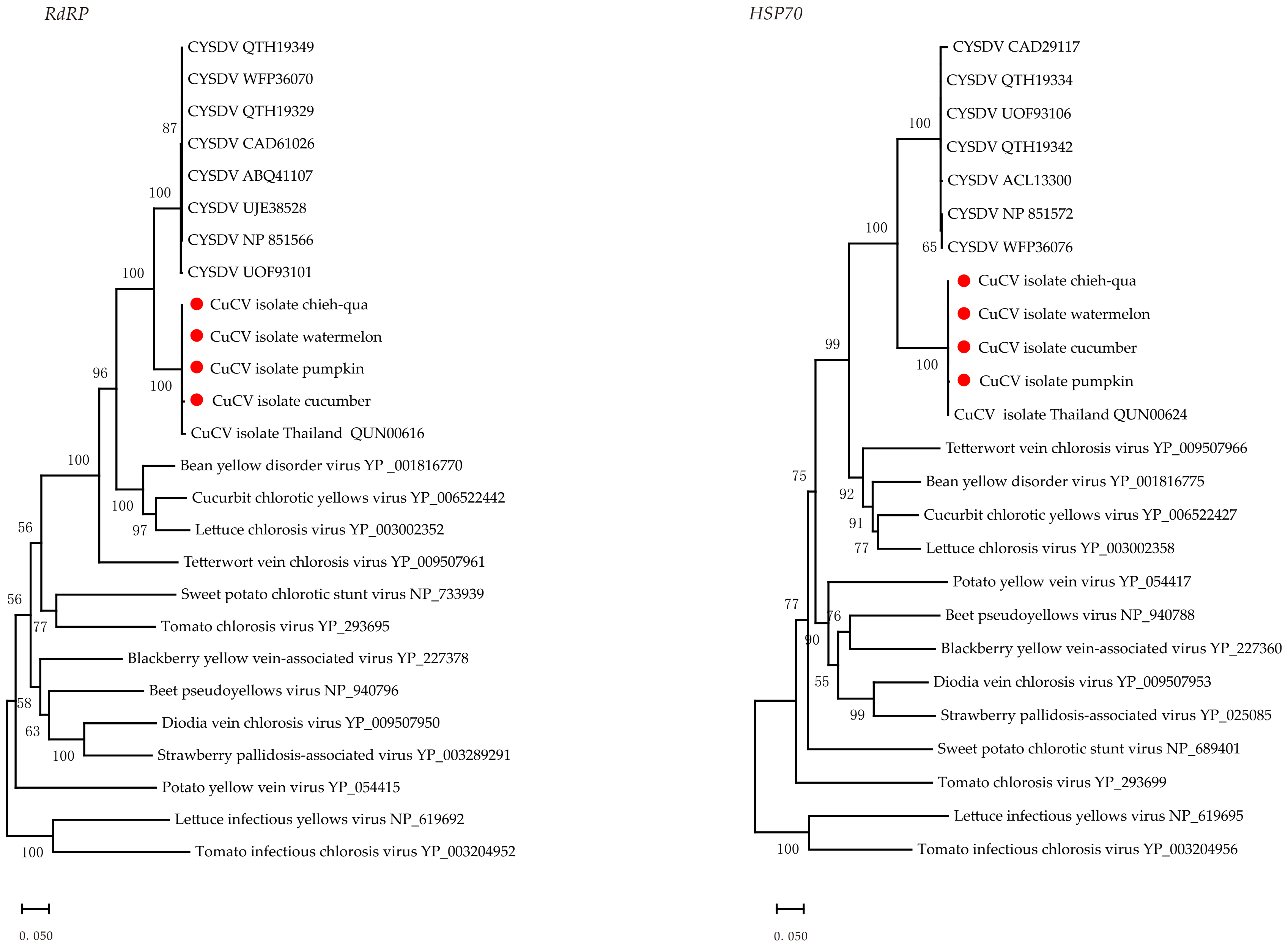

Figure S1. The Neighbor-joining (NJ) phylogenetic trees constructed on the RNA-dependent RNA polymerase (*RdRp*) and heat shock protein 70 (*HSP70*) gene nucleotide sequences of cucurbit chlorotic virus (CuCV), cucurbit yellow stunting disorder virus (CYSDV) and the representative members of the genus *Crinivirus*, family *Closteroviridae*.The newly identified viruses in this study are indicated by red dots.
