# Supplementary material for: Virome Profiling, New Virus Identification and the Prevalence and Distribution of Viruses Infecting Chieh-Qua (Benincasa hispida Cogn. var. chieh-qua How) in China
Source: Viruses. 2023 Jun 19;15(6):1396. doi: 10.3390/v15061396 (PMC10301065; doi:10.3390/v15061396)
Supplement: Supplementary file 1 [file viruses-15-01396-s001.zip › Table S1-10.pdf]

Table S1. Specific primers used to confirm the presence of the virus in the original plants and to survey virus prevalence

Virus	Accession No.	Primer[a]	Sequences (5'-3')	Expected Size(bp)	Annealing Temperature (°C)	Reference
cucurbit chlorotic virus (CuCV, genus *Crinivirus*, family *Closteroviridae*)	OP976048	CuCV-RNA1-F<br>CuCV-RNA1-R	CCTCTCATCCTCTTTTACCG<br>TAACCGTCACCATACCTAGT	550	53	this study
	OP976049	CuCV-RNA2-F<br>CuCV-RNA2-R	GTGGGCTAATTTGAGAGGAA<br>AAACGTGGTCTCTTAATGCT	528	55	this study
cucurbit chlorotic yellows virus (CCYV, genus *Crinivirus*, family *Closteroviridae*)	MH477611	CCYV-RNA1-F<br>CCYV-RNA1-R	TGTTAGATTTTCCGCTGACA<br>TACACTTTGTCCACCTTAGC	697	55	this study
watermelon silver mottle virus (WSMoV, genus *Orthotospovirus*, family *Tospoviridae*)	JX177647	WSMoV-L-F<br>WSMoV-L-R	ATAGAYGGGCARCCYTTCTT<br>AGRCTGCTTCTGARTGACCT	1001	55	this study
melon yellow spot virus (MYSV, genus *Orthotospovirus*, family *Tospoviridae*)	MH734110	MYSV-L-F<br>MYSV-L-R	TATYGGCATGGGRAAGAAAA<br>AGAATAGGAACCTTGCTTGG	732	55	this study
papaya ringspot virus (PRSV, genus *Potyvirus*, family *Potyviridae*)	AY027810	PRSV-F<br>PRSV-R	GCCTGAGCGATTRTAYARRG<br>CTGTCGATCRAARCTGTCHG	928	55	this study
chieh-qua endornavirus (CqEV genus *Alphaendornavirus*, family *Endornaviridae*)	OQ851472	CqEV-F<br>CqEV-R	TCCACAAGTAGCGGACCATT<br>AATGGGTGATCGGGTCCTTT	582	55	this study
cucumber mosaic virus (CMV genus *Cucumovirus*, family *Bromoviridae*)	OP204214	CP1<br>CP2	TTCGATAAGAAGCTTGTTTCGCG<br>GCCGTAAGCTGGATGGACAA	480	53	[8]
zucchini yellow mosaic virus (ZYMV, genus *Potyvirus*, family *Potyviridae*)	MN598579	ZP1<br>ZP2	TCAGGCACTCAGCCAACTGTG<br>TTACTGCATTGTATTCACACCTAGC	840	53	[8]
zucchini tigre mosaic virus (ZTMV, genus *Potyvirus*, family *Potyviridae*)	MF362994	ZTMVdF<br>ZTMVdR	GCATTAAGTTTACGACAGCGC<br>CAATCACTCTTGTCRGTGTC	632	53	[10]

[a] Two Primers (CuCV-RNA2-F and CuCV-RNA2-R) were only used in HTS validation, other primers were used in HTS validation and the prevalence survey of chieh-qua-associated virus

Y=C/T, R=A/G, H=A/C/T

Table S2. Primers used to complete the genomes of the new viruses

Virus	Primer	Sequences (5'-3')	Position	Expected Size (bp)	Annealing Temperature (°C)	Usage
cucurbit chlorotic virus (RNA1, OP976048)	CuCV-RNA1-GSP5	CTAAAAGCCCTAGGAGAGACTACG	320–297	320-	60	5′ terminal of RNA1
	UPM	CTAATACGACTCACTATAGGGCAA GCAGTGGTATCAACGCAGAGT	—			
	CuCV-RNA1-F1	GAAGAACTCTGACTACACAA	33–52	5842	51	complete genome of RNA1
	CuCV-RNA1-R1	GATAGGGTTGTCGTACTTCC	5874–5855			
	CuCV-RNA1-F2	GCTAAGGTTGACAGGTTGTA	5338–5357	3667	53	complete genome of RNA1
	CuCV-RNA1-R2	CTCAGAGACAGAAACTATAATCA	9004–8982			
	CuCV-RNA1-F3	GGTTTGGTTGTGAAAGCTTA	8845–8864	313	51	3′ terminal of RNA1
	anchored-dt-rev	TGTGTTGGGTGTGTTTGG	—			
cucurbit chlorotic virus (RNA2, OP976049)	CuCV-RNA2-GSP5	CATATTAATCTCAGTCCTCGATACC	438–414	172	57	5′ terminal of RNA2
	UPM	CTAATACGACTCACTATAGGGCAA GCAGTGGTATCAACGCAGAGT	—			
	CuCV-RNA1-F1	TGGTATCGAGGACTGAGATT	147–166	3698	53	complete genome of RNA2
	CuCV-RNA1-R1	GTGGTGGAATATTGGACTGT	3844–3825			
	CuCV-RNA1-F2	AACCTGAGATGCAACATCTT	2541–2560	5230	53	complete genome of RNA2
	CuCV-RNA1-R2	ACTAAGTTCTCAAGGTGTACC	7770–7750			
	CuCV-RNA2-F3	ACGGATTTGGTAAGAACACT	7339–7358	450	51	3′ terminal of RNA2
	anchored-dt-rev	TGTGTTGGGTGTGTTTGG	—			

Table S3. RT-PCR confirmation of the presence of the virus in individual plants

**Virus**	**CS-1**	**CS-15**	**CS-16**	**CS-37**	**CS-78**
cucurbit chlorotic virus	-	+	-	+	-
cucurbit chlorotic yellows virus	+	+	-	+	-
watermelon silver mottle virus	+	+	-	-	-
melon yellow spot virus	+	+	+	+	+
papaya ringspot virus	-	+	-	-	-
chieh-qua endornavirus	-	+	+	-	-

"+" indicated virus was detected in the sample, "-" indicated no virus was detected in the sample. The presence of cucurbit chlorotic virus, cucurbit chlorotic yellows virus, watermelon silver mottle virus, melon yellow spot virus, papaya ringspot virus, and chieh-qua endornavirus were confirm by specific primers CuCV-RNA1-F/CuCV-RNA1-R and CuCV-RNA2-F/CuCV-RNA2-R, CCYV-RNA1-F/CCYV-RNA1-R, WSMoV-L-F/WSMoV-L-R, MYSV-L-F/MYSV-L-R, PRSV-F/PRSV-R, and CqEV-F/CqEV-R in Table S1, respectively.

Table S4. The percent amino acid identity RdRP, HSP70, and CP between the cucurbit chlorotic virus (CuCV) and other unclassified and formal members in the genus *Crinivirus* of the family *Closteroviridae*

**Virus**	**Accession**	**RdRP**	**HSP70**	**CP**
cucurbit yellow stunting disorder virus	YP_006522442, YP_006522427, YP_006522431	90.10	83.91	74.1
lettuce chlorosis virus	YP_003002352, YP_003002358, YP_003002362	78.42	73.78	60.82
bean yellow disorder virus	YP_001816779, YP_001816775, YP_001816770	79.41	73.42	62.04
cucurbit chlorotic yellows virus	YP_006522442, YP_006522427, YP_006522431	78.22	73.78	64.08
tetterwort vein chlorosis virus	YP_009507961, YP_009507966, YP_009507970	73.86	69.80	62.92
blackberry yellow vein-associated virus	YP_227378, YP_227360, YP_227364	63.96	63.47	40.66
tomato chlorosis virus	YP_293695, YP_293699, YP_293703	62.10	58.23	34.89
beet pseudoyellows virus	NP_940796, NP_940788, NP_940792	60.79	62.21	37.55
strawberry pallidosis-associated virus	YP_003289291, YP_025085, YP_025089	60.00	62.03	33.47
sweet potato chlorotic stunt virus	NP_733939, NP_689401, NP_689404	59.21	60.94	33.06
diodia vein chlorosis virus	YP_009507950, YP_009507953, YP_009507957	58.02	63.47	41.32
potato yellow vein virus	YP_054415, YP_054417, YP_054421	55.84	61.48	41.49
lettuce infectious yellows virus	NP_619692, NP_619695, NP_619697	52.87	49.28	26.16
tomato infectious chlorosis virus	YP_003204952, YP_003204956, YP_003204960	50.69	52.98	26.12

Table S5. Overall nucleotide sequence identity (%) between chieh-qua endornavirus (CqEV) and members of different species in the genus *Alphaendornavirus* of the family *Endornaviridae*.

Virus	Accession	Identity (%)
cucumis melo endornavirus	KT727022	65.41
lagenaria siceraria endornavirus	KF562072	55.01
persea americana endornavirus 1	JN880414	48.59
yerba mate endornavirus	KJ634409	50.89
oryza rufipogon endornavirus	AB014344	47.83
oryza sativa endornavirus	D32136	48.80
basella alba endornavirus 1	AB844264	45.82
phaseolus vulgaris endornavirus 1	KT456287	46.20
phytophthora endornavirus 1	AJ877914	38.55
phaseolus vulgaris endornavirus 3	MG242064	36.61
agaricus bisporus endornavirus 1	KY357509	38.01
rhizoctonia solani endornavirus 2	KT823701	36.91
bell pepper endornavirus	KX977569	35.30
hot pepper endornavirus	KR080326	35.40
phaseolus vulgaris endornavirus 2	KT456288	34.94
winged bean endornavirus 1	LC144945	34.95
cluster bean endornavirus	MG764084	36.29
rhizoctonia cerealis endornavirus 1	KF311065	35.09
helianthus annuus endornavirus	MF362666	36.44
erysiphe cichoracearum endornavirus	KT388110	34.83
grapevine endophyte endornavirus	JX678977	33.80
helicobasidium mompa endornavirus 1	AB218287	33.87
hordeum vulgare endornavirus	KT721705	35.71
vicia faba endornavirus	AJ000929	34.93

Table S6. Occurrence of cucurbit chlorotic virus (CuCV) in different hosts

Location	Percentage of Positive Samples (No. of Virus Infected Samples/ Total no. of Plants Analyzed)		
	**Pumpkin**	**Watermelon**	**Cucumber**
Chengmai	100 (2/2)	-	0 (0/5)
Wenchang	0 (0/10)	0 (0/0)	0 (0/4)
Ding'an	-	-	40 (2/5)
Lingshui	-	16.67 (1/6)	0 (0/9)
Sanya	40 (2/5)	14.29 (1/7)	38.46 (5/13)
Dongfang	0 (0/6)	22.22 (2/9)	14.29 (1/7)
Danzhou	-	-	0 (0/6)
Total	27.39 (4/23)	15.38 (4/26)	16.33 (8/49)

"-" indicated no samples were collected in this region

Table S7. The pairwise nucleotide identities (%) of genome RNA1 among cucurbit chlorotic virus (CuCV) virus and cucurbit yellow stunting disorder virus (CYSDV) isolates available in the NCBI database.

**Virus abbreviations**	**Isolates**	**Accession**	**Chieh-qua-CM**	**Thailand**	**AlLM**	**14C**	**Arizona**	**CF2A-524**	**CaF2A2019**	**Spanish**	**DSMZ_PV-1144**
CuCV	Chieh-qua-CM	OP976048	100								
CuCV	Thailand	MT813029	99.18	100							
CYSDV	AlLM	AY242077	65.62	65.51	100						
CYSDV	14C	MW147553	65.74	65.65	99.42	100					
CYSDV	Arizona	EF547827	65.79	65.70	99.48	99.81	100				
CYSDV	CF2A-524	MW685457	65.71	65.62	99.52	99.75	99.81	100			
CYSDV	CaF2A2019	MW685460	65.76	65.65	99.55	99.74	99.80	99.77	100		
CYSDV	Spanish	AJ537493	65.70	65.62	99.53	99.51	99.56	99.48	99.58	100	
CYSDV	DSMZ_PV-1144	OL584359	65.67	65.62	99.27	99.33	99.4	99.34	99.42	99.34	100

Table S8. The pairwise nucleotide identities (%) of genome RNA2 among cucurbit chlorotic virus (CuCV) virus and cucurbit yellow stunting disorder virus (CYSDV) isolates available in the NCBI database.

**Virus abbreviations**	Isolates	Accession	Chieh-qua-CM	Thailand	AlLM	Arizona-1	CaF2A-1	CaF2A-2	DSMZ_PV-1144	Spanish
CuCV	Chieh-qua-CM	OP976049	100							
CuCV	Thailand	MT819949	99.35	100						
CYSDV	AlLM	AY242078	67.44	67.46	100					
CYSDV	Arizona-1	FJ492808	67.46	67.49	99.80	100				
CYSDV	CaF2A-1	MW685458	69.00	69.04	99.80	99.79	100			
CYSDV	CaF2A-2	MW685459	69.04	69.09	99.79	99.77	99.89	100		
CYSDV	DSMZ_PV-1144	OL584360	67.29	67.29	99.56	99.48	99.51	99.50	100	
CYSDV	Spanish	AJ439690	67.30	67.33	99.54	99.41	99.33	99.32	99.17	100

Table S9. The percent amino acid identity of RdRp (lower left) and HSP70h (upper right) among the formal species in the genus *Crinivirus* of the family *Closteroviridae*.

	**LIYV**	**TICV**	**SPCSV**	**ToCV**	**PYVV**	**TwVCV**	**CYSDV**	**BYDV**	**LCV**	**DVCV**	**SPaV**	**BPYV**	**BYVaV**
LIYV	100	63.83	50.81	50.63	49.91	50.72	49.64	49.01	47.74	51.36	51.44	48.82	48.56
TICV	62.57	100	52.89	53.43	53.79	53.07	53.16	54.51	53.43	52.17	52.53	52.71	53.07
SPCSV	55.45	53.27	100	60.83	61.55	66.06	61.84	63.72	63.36	64.26	63.72	63.54	63.72
ToCV	55.75	55.75	66.67	100	57.58	59.93	60.22	61.01	59.57	59.39	60.65	59.75	58.66
PYVV	53.47	56.44	59.80	60.71	100	62.27	61.84	64.26	64.26	64.26	64.98	67.51	67.51
TwVCV	53.27	51.88	61.39	61.71	56.83	100	71.43	77.52	78.96	67.45	64.93	63.18	63.60
CYSDV	54.44	51.09	60.40	62.90	55.84	74.26	100	73.60	73.24	65.28	63.11	62.57	64.01
BYDV	54.58	51.49	58.22	61.51	57.03	76.63	79.49	100	83.81	66.73	66.13	64.44	63.54
LCV	54.65	50.50	57.43	59.92	55.45	74.06	76.63	85.74	100	65.47	66.13	64.98	63.18
DVCV	53.27	53.66	62.18	58.73	62.77	61.39	60.40	58.02	59.41	100	80.72	69.31	68.41
SPaV	54.65	54.46	63.17	58.73	60.99	60.99	60.20	59.01	59.21	77.03	100	72.74	70.09
BPYV	55.64	55.25	61.78	62.70	63.56	60.79	60.99	59.80	61.39	69.11	68.71	100	72.38
BYVaV	57.03	55.84	62.57	63.10	63.56	64.36	63.76	62.97	63.96	69.11	70.10	70.89	100

The values of the amino acid identities greater than 75% are shaded in red. The full names of the criniviruses are listed below: lettuce infectious yellows virus (LIYV, NP_619692 and NP_619695), tomato infectious chlorosis virus (TICV, YP_003204952 and YP_003204956), sweet potato chlorotic stunt virus (SPCSV, NP_733939 and NP_689401), tomato chlorosis virus (ToCV, YP_293695 and YP_293699), potato yellow vein virus (PYVV, YP_054415 and YP_054417), tetterwort vein chlorosis virus (TwVCV, YP_009507961 and YP_009507966), cucurbit yellow stunting disorder virus (CYSDV, YP_006522442 and YP_006522427), bean yellow disorder virus (BYDV, YP_001816779 and YP_001816775), lettuce chlorosis virus (LCV, YP_003002352 and YP_003002358), diodia vein chlorosis virus (DVCV, YP_009507950 and YP_009507953), strawberry pallidosis-associated virus (SPaV, YP_003289291 and YP_025085), beet pseudoyellows virus (BPYV, NP_940796 and NP_940788), blackberry yellow vein-associated virus (BYVaV, YP_227378 and YP_227360).

Table S10. Percentages of the nucleotide (Nt) and deduced protein sequence (AA) identities between CuCV (RNA1: OP976048, OP976050, OP976052, OP976054, MT813029; RNA2: OP976049, OP976051, OP976053, OP976055 and MT819949) and CYSDV (RNA1: MW147553, EF547827, MW685457, MW685460, AY242077, AJ537493 and OL584359; RNA2: FJ492808, AY242078, MW685458, MW685459, OL584360 and AJ439690) available in the NCBI database

	RNA1							RNA2									
	5′-UTR	ORF1a	RdRp	p5	p25	p22	3′-UTR	5′-UTR	p4	HSP70	p6	p59	p9	CP	CPm	p26	3′-UTR
AA	N/A	64.79~66.21	89.5~90.3	N/A	33.33~34.3	44.79~46.88	N/A	N/A	N/A	82.64~84.09	48.15~50	70.08~71.43	51.9~53.16	72.51~74.50	55.77~56.64	70.18~71.49	N/A
Nt	N/A	65.73/66.17	76.61~77.21	N/A	49.92~50.57	54.13~55.18	59.8~65.88	N/A	N/A	74.49~74.97	60~61.21	67.95~68.66	58.75~59.17	71.89~74.56	64.14~64.51	68.41~69.58	57.22~61.03
